# FF-DEIM: DEIM with Image Dehazing and Self-Supervised Pretraining for Catenary Support Component Detection

**DOI:** 10.3390/s26155000

**Published:** 2026-08-06

**Authors:** Lingzhi Zhang, Jinyong Huang, Guojin Qin, Jincheng Cao, Fei Fan, Hui Wang, Haonan Yang

**Affiliations:** 1 Hunan Provincial Engineering Technology Research Center for High-Speed Railway Operation Safety Assurance, Hunan Railway Professional Technology College, Zhuzhou 412001, China; zlz13789099952@163.com (L.Z.);; 2 China Railway No. 5 Engineering Group Co., Ltd., Changsha 410017, China; 3 School of Electrical Engineering, Southwest Jiaotong University, Chengdu 610097, China

**Keywords:** deep learning, object detection, catenary support components (CSCs), self-supervised learning

## Abstract

The catenary support component (CSC) is a key part of the electrified railway system, and its operational status directly affects railway operational safety. These components’ images are collected using inspection equipment and detected using computer vision techniques. However, catenary network inspection faces the following issues: (1) due to limitations in the equipment’s shooting angle and changes in viewing distance, the collected images contain multi-scale and multi-class problems, and (2) the railway environment is highly variable, and adverse weather conditions such as fog, rain, and low light affect the imaging devices, leading to degraded image quality. To address these issues, this paper proposes a novel detection framework, FF-DEIM, for detecting catenary support components. First, a dual-channel fusion network (DCFNet) is introduced, which significantly improves image quality by removing foreground interferences such as fog, raindrops, and dynamic blur. Second, a pretraining framework based on contrastive learning, mask image modeling with contrastive learning (MIMCL), is designed to enhance the model’s focus on key regions of the catenary network components, optimizing feature extraction capabilities and improving model convergence speed. Then, a feature-focusing pyramid network (FFPN) is proposed, which uses the focus feature module to fuse cross-level contextual features, enhancing the ability to capture local details and improving the model’s small object detection performance. Finally, a drone-based catenary network image dataset, including scenes with fog, rain, and low light, is constructed, and experiments validate the effectiveness of the proposed method.

## 1. Introduction

The catenary network is a crucial component of electrified railways, responsible for continuously supplying power to trains [1,2,3,4,5]. However, during high-speed operation, the frequent mechanical contact between the pantograph and the catenary network can cause strong vibrations, potentially leading to structural failures such as loosening, damage, or breakage of the catenary support component (CSC) system [6,7,8,9,10]. If these issues are not detected in time, they pose a serious threat to train operation safety and may even lead to major accidents. For a long time, catenary network inspections have mainly relied on manual methods, which, although ensuring a certain level of accuracy, are labor-intensive and inefficient [11,12]. Additionally, due to human factors, inconsistent or missed inspections may occur. To improve detection efficiency and accuracy, the Chinese electrified railway system has started using non-contact inspection equipment such as the JX300 and unmanned aerial vehicle (UAV) to collect catenary network images, enabling more efficient inspection tasks [13,14]. However, the collected images face two main issues due to limited equipment shooting angles, varying view distances, and changing environmental conditions (such as fog, rain, and low light): (1) there are significant size differences between different components (e.g., screws, insulators, and brackets have noticeable scale differences, and small parts like pins are too tiny), and (2) images contain phenomena from fog, rain, and low light scenes.

Existing catenary network detection methods can be divided into traditional methods and deep learning-based methods. Traditional methods, such as the one proposed by Yang et al., use affine invariant moment detection to inspect catenary network insulators, classifying anomalies through Hough transform and grayscale statistical curves [15]. The same team later proposed a defect detection method for catenary network insulators based on SURF feature matching, which quickly and accurately locates defective insulators through feature matching, angle correction, and grayscale statistics [16]. While traditional methods can offer high accuracy and low computational cost for specific tasks, they rely on manually designed features and perform poorly under complex environments and scale variations [17]. With the development of deep learning technology, deep learning-based algorithms have gradually become the focus of catenary network detection research [18]. These methods overcome the limitations of traditional methods by automatically learning image features, especially in multi-faceted complex environments and addressing multi-scale issues, demonstrating stronger robustness and accuracy [19,20]. It is worth noting that most of these methods focus on region-level components but lack research on multi-scale components in global images. To address this, the CSCNET framework proposed by Liu et al. effectively achieves precise positioning of the CSC through cascading coarse and fine localization networks [21]. In practical applications, the collected images often face severe visual degradation issues. Liu et al. proposed an improved Faster R-CNN method that solves the small-scale detection problem of support sleeve bolts [22]. However, these methods typically use feature concatenation to obtain multi-scale information, lacking exploration of cross-scale feature information. Therefore, this paper adopts a cross-layer focusing approach to extend cross-layer feature information to all feature layers to address this problem. Furthermore, catenary network images are often disturbed by complex backgrounds, such as the surrounding environment and rails, making object detection more difficult. Research has shown that unsupervised learning methods can effectively separate the foreground from the background, reducing background interference. Contrastive learning focuses on the identification of local defects and details. Hu proposed a self-supervised steel surface defect detection model that learns defect features on unlabeled data through contrastive learning [23]. Mask image modeling focuses on global structure and the relationships between components [24]. Contrastive learning may not effectively handle global structural information, while mask learning may not perform well on images with complex backgrounds and high noise [25]. Factors such as lighting changes, rain, and fog can still cause images to have low contrast, blurred edges, and color deviations, severely affecting the accuracy of subsequent object detection tasks.

Traditional dehazing methods are typically based on the RGB color space for image processing, directly recovering the image’s brightness and color. For example, Fattal proposed a single image dehazing method based on color lines, analyzing the 1D distribution in the RGB color space to recover the scene transmission map and using a Markov random field model to solve transmission issues in isolated areas [26]. Additionally, dehazing methods based on the YCbCr color space, especially for hazy images, better preserve image structure and color perception, improving dehazing effects. For instance, Saihood proposed a YCbCr color space-based dehazing method combining Sigmoid function mapping, adaptive histogram equalization (AHE), and dark channel prior (DCP) to optimize brightness (Y channel) and chrominance (CbCr channels) [27]. While the RGB color space is intuitive, its coupling of brightness and chrominance information limits the effectiveness of dehazing in complex haze environments. In contrast, the YCbCr color space enhances image quality by separating brightness (Y channel) and chrominance (CbCr channels), although it is more complex to operate [28]. Therefore, Zahid Tufail combined the DCP method with RGB and YCbCr color spaces by calculating transmission maps in both and using dark channel information to estimate atmospheric light and optimize edge information [29]. However, traditional methods generally have limitations when handling complex scenes, detail recovery, and high dynamic range images. To overcome these challenges, deep learning methods have been widely applied to image dehazing tasks, using neural networks to automatically learn image features, significantly improving dehazing effects and detail recovery capabilities. For example, Ki et al. proposed the conditional BEGAN method, using a U-Net generator and RGB input images to increase the receptive field and optimize the discriminator using Wasserstein distance [30]. Despite several dehazing networks achieving good results, the advantages of combining RGB and YCbCr color spaces in deep learning have not been fully explored. Most methods focus primarily on a single-color space, either RGB or YCbCr, without fully combining their complementary advantages during training [31]. Transfer learning is often limited by domain gap issues, where pre-trained knowledge from source datasets may not effectively transfer to target-specific scenarios, leading to suboptimal feature adaptation and performance degradation [32].

Recent studies address multi-scale and degradation issues through improved feature pyramid designs. Transformer-based multi-scale modeling and deep image restoration techniques are used, such as dehazing and low-light enhancement [33]. Related advances in industrial defect detection and interpretable machine learning have also provided valuable insights into feature representation, model robustness, and reliable decision-making [34,35]. Beyond conventional visual perception, the integration of computational modeling with intelligent design and automated manufacturing has demonstrated the value of constructing coordinated and automated processing frameworks [36]. AIoT-based research has further combined secure trajectory prediction with task offloading, highlighting the importance of jointly considering intelligent prediction, computational efficiency, and system reliability in safety-critical applications [37]. In complex visual environments, adaptive cross-attention mechanisms have been employed to strengthen complementary information interaction between multispectral modalities [38]. Multivariate feature learning and associative spatial information enhancement have also been investigated to improve object representation under adverse weather conditions, such as snowy scenes [39]. Moreover, large-kernel spatially parallel feature fusion has shown the potential to enhance spatial-context aggregation and multi-level representation for monocular 3D perception [40]. These studies collectively indicate that coordinated computation, cross-modal interaction, spatial information enhancement, and robust feature fusion are essential for reliable perception in complex environments. However, most existing methods treat multi-scale variation, image degradation, and robust feature learning as separate problems, leading to suboptimal feature consistency between image enhancement and object detection. Therefore, there is still a lack of a unified framework that can simultaneously handle multi-scale variation, image degradation, and robust feature representation for CSC detection in complex railway environments.

Inspired by the above analysis, this paper designs a new detector (FF-DEIM) for CSC detection in complex and variable environments. Specifically, the main contributions of this paper are as follows:

(1) A dual-channel fusion network (DCFNet) image dehazing method is proposed, which significantly improves the quality of input images by removing foreground interferences (such as fog, raindrops, and dynamic blur).
(2) A pretraining method based on contrastive learning for mask image modeling (MIMCL) is designed, which enhances the model’s ability to focus on key regions of the catenary network components, optimizing the feature extraction ability of the backbone and improving overall detection performance and model convergence speed.
(3) A feature-focusing pyramid network (FFPN) module is proposed, which uses the focus feature module to fuse cross-layer contextual features, enhancing the ability to capture local details and improving the detection performance of multi-scale components.
(4) A UAV-based catenary network image dataset is constructed, which includes images with fog, rain, and low light conditions. Various experiments demonstrate the effectiveness of the proposed method.

The following content is structured as follows: Section 2 introduces the proposed method. Section 3 mainly introduces the experimental environment and settings, datasets, experimental results, and analysis of this paper. Section 4 presents the conclusions and further prospects.

## 2. The Proposed Method

**Figure 1:**
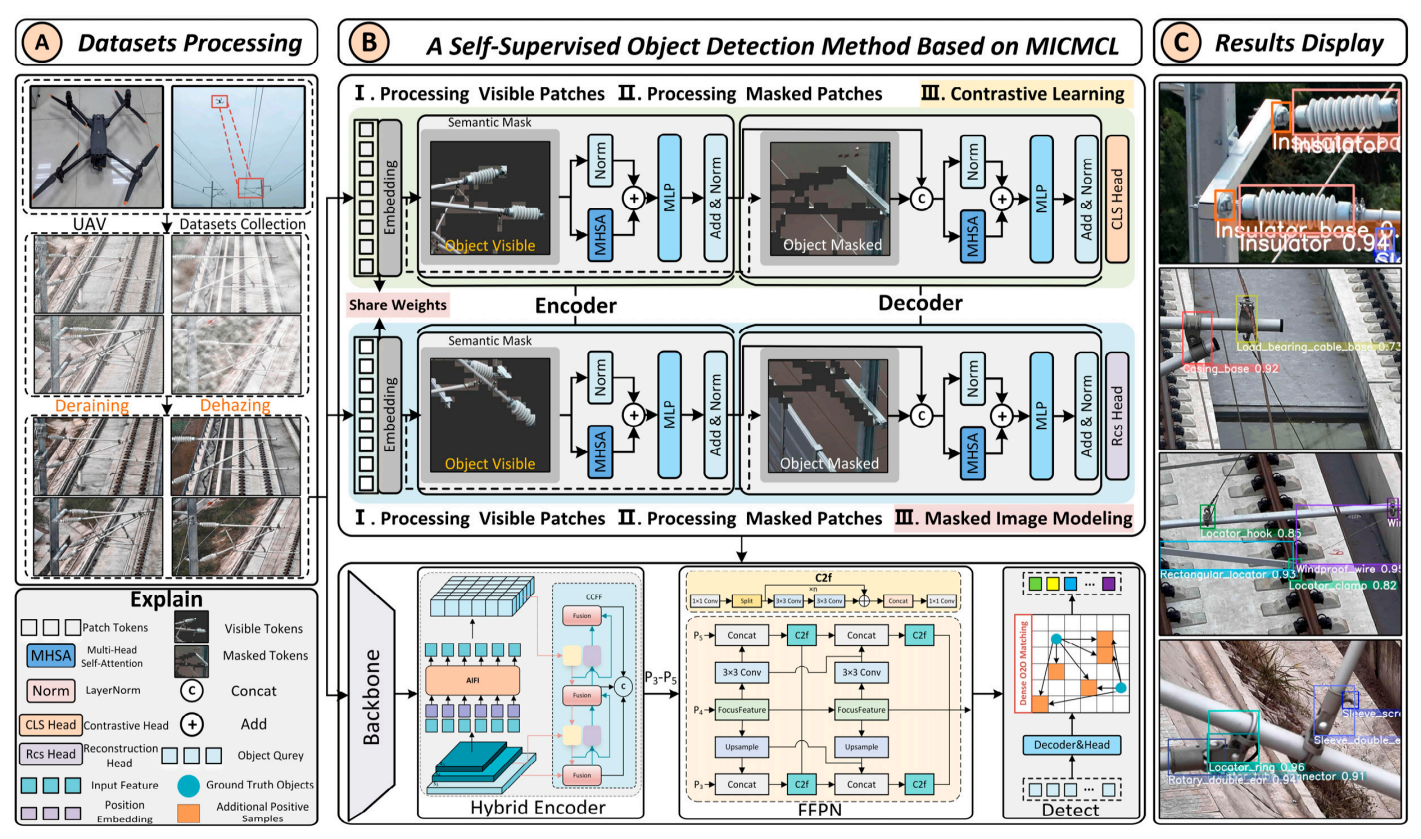
The structural diagram of FF-DEIM. The overall process involves image processing to remove interference, pretraining the images using MIMCL, and finally using FF-DEIM for detection.

To address image degradation, large-scale variation, and weak-feature representation in CSC detection, this study proposes a three-stage framework. The design follows a progressive feature refinement strategy, where image quality enhancement, representation learning, and object detection are jointly optimized in a coarse-to-fine manner. As shown in Figure 1, the first stage employs a DCFNet to improve input image quality by combining RGB and YCbCr feature representations, which helps suppress weather-related degradation while preserving structural details. The second stage introduces masked image modeling with MIMCL to learn robust feature representations by integrating global context modeling and local discrimination under limited annotations. In the final stage, DETR is enhanced with an FFPN to strengthen multi-scale feature representation and improve small object detection before transformer-based decoding.

### 2.1. Guided Image Dehazing with Dual-Color Feature Fusion

**Figure 2:**
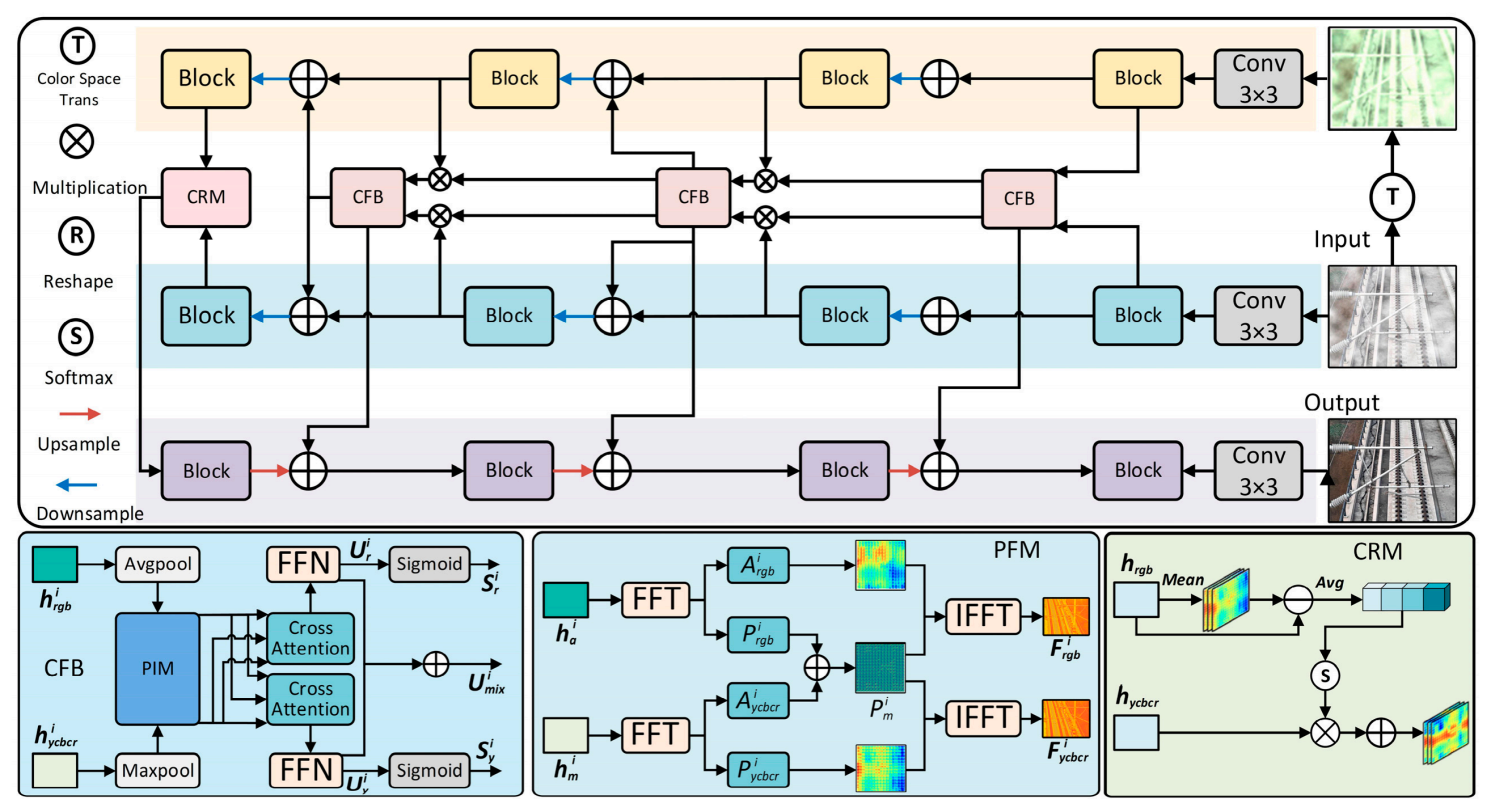
The structural diagram of DCFNet.

We present a DCFNet designed to tackle the challenging task of image dehazing in real-world scenarios. DCFNet utilizes both the RGB and YCbCr color spaces to enhance image texture details and color perception. The architecture, as illustrated in Figure 2, adopts an asymmetric encoder–decoder structure with large kernel attention blocks, ensuring that both coarse structures and fine details are preserved during the dehazing process. The process begins by converting the input hazy image from the RGB color space to the YCbCr color space using a standard linear transformation. After this transformation, both RGB and YCbCr versions of the image are processed through a shared encoder to extract multi-scale feature representations, capturing both global and local image features. These features are then processed by the chromatic fusion bridge (CFB), which consists of two key modules: the progressive fusion module (PFM) and the adaptive cross-attention module (ACAM). For the RGB features, an average pooling operation is applied to smooth out noise and preserve the global structure of the image.

(1) $$h_{a}^{i}=AvgPool\left(h_{rgb}^{i}\right)$$

where $h_{rgb}^{i}$ is the input RGB feature map, and $h_{a}^{i}$ is the feature map after the average pooling operation, which retains the global structure of the image by smoothing out noise. Similarly, for the YCbCr features, a max pooling operation is applied to enhance local image details and chrominance, ensuring robust activation values:

(2) $$h_{m}^{i}=MaxPool\left(h_{ycbcr}^{i}\right)$$

where $h_{ycbcr}^{i}$ is the input YCbCr feature map, and $h_{m}^{i}$ is the feature map after the max pooling operation, which enhances local details and chrominance, making them more robust to minor variations. In the progressive fusion module (PFM), the amplitude and phase components of the RGB and YCbCr features are extracted using Fourier transform (FFT). The amplitude captures the overall intensity, while the phase retains important structural information.

(3) $$\begin{array}{c} A_{rgb}^{i},P_{rgb}^{i}=S\left(F\left(f_{rgb}^{i}\right)\right)\\ A_{ycbcr}^{i},P_{ycbcr}^{i}=S\left(F\left(f_{ycbcr}^{i}\right)\right) \end{array}$$

where *F(·)* represents the Fourier transform operation, and *S(·)* is the phase and amplitude extraction. $A_{rgb}^{i}$ and $P_{ycbr}^{i}$ are the amplitude and phase components of the RGB feature, while $A_{ycbcr}^{i}$ and $P_{ycbcr}^{i}$ are the amplitude and phase components of the YCbCr feature. The phase components from both color spaces are then fused to enhance the image’s structural clarity:

(4) $$P_{m}^{i}=Conv_{3\times 3}\left(P_{rgb}^{i}\right)+Conv_{3\times 3}\left(P_{ycbcr}^{i}\right)$$

where *conv*_3×3_ is a convolution operation applied to merge the phase components from both color spaces, $P_{rgb}^{i}$ and $P_{ycbcr}^{i}$, to form a combined phase spectrum $P_{m}^{i}$ that helps enhance structural clarity. Finally, the fused amplitude and phase components are reconstructed into the spatial domain via the inverse Fourier transform (IFFT):

(5) $$\begin{array}{c} F_{rgb}^{i}=F^{-1}\left(Conv_{1\times 1}\left(A_{rgb}^{i}\right),P_{m}^{i}\right)\\ F_{ycbcr}^{i}=F^{-1}\left(Conv_{1\times 1}\left(A_{ycbcr}^{i}\right),P_{m}^{i}\right) \end{array}$$

where *F^−^*^1^*(·)* represents the inverse FFT. Next, the adaptive cross-attention module (ACAM) further refines the important regions in the RGB and YCbCr features using a cross-attention mechanism. We first downsample $A_{rgb}^{i}$ and $A_{ycbcr}^{i}$ to obtain $U_{r}^{i}$ and $U_{y}^{i}$. The refined features are passed to the decoder through residual connections, resulting in the final fused feature.

(6) $$U_{mix}^{i}=Sig\left(U_{r}^{i}\right)⊙h_{rgb}^{i+1}+Sig\left(U_{y}^{i}\right)⊙h_{ycbcr}^{i+1}$$

where *Sig* (*·*) is an activation function, and ⊙ denotes element-wise multiplication. The final fused feature map $U_{mix}^{i}$ is the result of the attention-guided fusion of the RGB and YCbCr features. Finally, after processing both RGB and YCbCr features, DCFNet uses the color enhancement module (CEM) to improve color perception. The enhanced chrominance information is fused into the RGB features.

(7) $$D_{o}=v_{c}⊙h_{rgb}^{i}+h_{ycbcr}^{i}$$

where $v_{c}$ is the attention weight calculated from the YCbCr features and *D_o_* is the final enhanced RGB feature with improved color perception. To optimize the network’s performance, DCFNet employs a multi-scale loss function during training. This combines pixel-level L1 loss, structural similarity index (SSIM) loss, and FFT. The learning strategies of this module can be introduced in Section 2.4.

### 2.2. Masked Image Modeling with Contrastive Learning

As introduced in the Introduction, contrastive learning (CL) tends to focus on fine-grained features such as local defects or geometric details of key components in catenary systems, enhancing the model’s discriminative ability for localized targets [41]. In contrast, masked image modeling (MIM) emphasizes the understanding of the global structure and interrelationships among components, thereby offering stronger generalization capabilities. This contrast highlights a fundamental trade-off in unsupervised pretraining between local discriminability and global generalization. To bridge this gap, we employ a contrastive mask learning method, which combines the principles of MIM and CL. The method works by masking parts of the input image, creating visible and masked regions, and training the model to predict the masked portions based on the visible ones. The objective is to guide the model to learn valuable feature representations that focus on visible image parts, while simultaneously distinguishing between the unmasked and masked areas. To further enhance the semantic relevance of masked modeling, we incorporate semantic masks as guidance during the patch masking process [42,43]. Unlike purely random masking, we selectively mask regions corresponding to catenary components and divide the image into foreground and background masks. These are subsequently treated as visible and masked patches, respectively, guiding the model to focus more effectively on the reconstruction of critical structural information.

**Figure 3:**
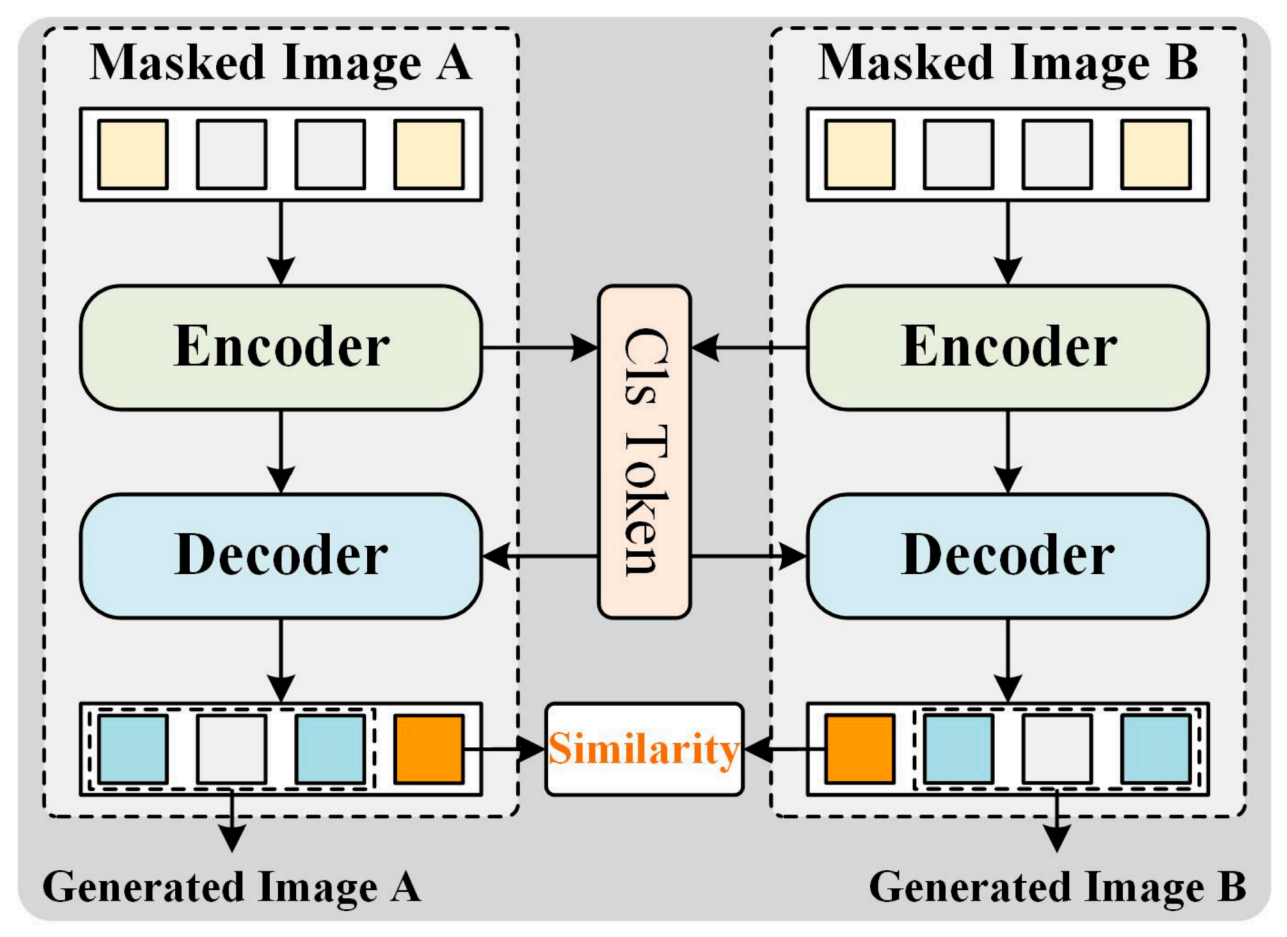
The structural diagram of MIMCL.

In this pretraining stage, as shown in Figure 3, the input image is divided into visible patches *P_v_* and masked patches *P_m_*. The encoder processes the visible patches $P_{v}$ to generate latent representations *L_v_*, capturing essential visual information. The decoder then uses these representations along with a class token *T_cls_* to reconstruct the masked regions Pm. This process is done by passing through multiple transformer blocks that progressively refine the predicted masked patches. We use a vision transformer (ViT) as the backbone of our model due to its effectiveness in capturing long-range dependencies within image patches. ViT processes images by dividing them into non-overlapping patches, which are then linearly embedded into fixed-size vectors. The advantage of ViT lies in its ability to model global relationships through self-attention mechanisms, making it well-suited for our pretraining task. The semantic masks in MIMCL are generated automatically from the teacher network’s patch-level attention responses. The normalized attention scores are thresholded to separate foreground and background patches without using bounding boxes, segmentation masks, category labels, or other manual annotations. Pretraining uses only unlabeled training images, excluding validation and test data, thereby preserving the self-supervised setting and preventing data leakage.

The encoder transforms the visible patches *P_v_* into latent representations $L_{v}$. In the first step, the encoder linearly projects each visible patch into embeddings. To retain spatial information, position embeddings *X_v_* are added to these embeddings. These embeddings are then processed through multiple transformer layers, using self-attention mechanisms to generate the latent representations $L_{v}$, which encapsulate the key features of the visible patches. The decoder, which plays a dual role, takes these latent representations and transforms them. Its primary function is to convert the latent representations of both visible patches *P_v_* and masked patches Pm back into the reconstructed masked patches. This transformation involves passing through several transformer layers, followed by a linear layer that accurately regenerates the original masked patches. In addition, the decoder refines the input class token $Z_{cls}$ into an enhanced version *Z_cls_*, which is crucial for effective contrastive learning.

Unlike traditional methods, where the class token is integrated early in the process, our method strategically delays the integration of the class token until the decoding phase. This allows the encoder to focus more on extracting a diverse range of visual features from the visible patches, while the decoder fine-tunes the class token, balancing the model’s objectives of feature extraction and classification. During the unsupervised pretraining phase, we begin by sampling an input image from a large, unlabeled base dataset. To generate two distinct views of the image, we apply random augmentations, resulting in two different versions, I_1_ and I_2_. These augmented views are processed separately by two networks: a teacher network, parameterized by $\theta_{t}$, and a student network, parameterized by *θ_s_*. The key idea behind this approach is that the teacher network is updated through an exponentially moving average (EMA) of the student network, which facilitates a more stable and consistent knowledge transfer between the two networks. The learning strategies of this module can be introduced in Section 2.4.

### 2.3. DETR with Feature-Focused Pyramids and Matching-Aware Design

In this work, we employ DETR with feature-focused pyramids and matching-aware design (FF-DEIM) for localization tasks on de-hazed and de-rained images, leveraging pre-trained weights from MIMCL to fine-tune the model for enhanced performance. DEIM, a variant of DETR, shares similarities with the traditional transformer-based architecture but introduces several key improvements to optimize matching efficiency and training convergence. The DEIM architecture builds upon the standard transformer design, utilizing a backbone encoder for feature extraction, followed by an efficient encoder to enhance representation learning. The FFPN module then refines features across multiple scales, improving multi-scale feature representations. Finally, the decoder processes these features to output high-quality detection results.

**Figure 4:**
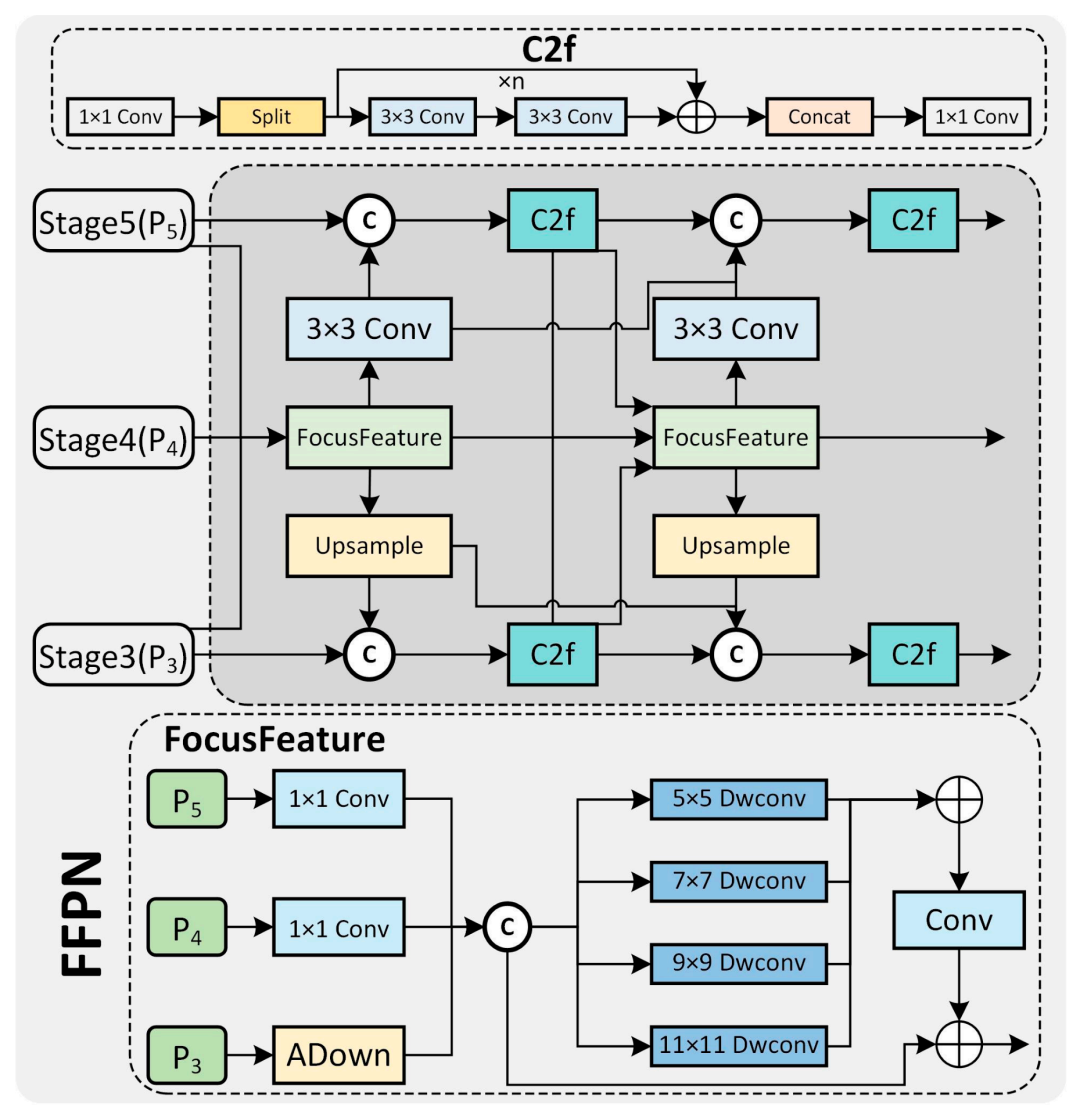
The structural diagram of FFPN.

The FFPN module is illustrated in Figure 4, which is designed to refine features across multiple scales, improving the representation of both local and global information in the input image. The module begins by projecting the input feature maps into a unified hidden dimension through a 1 × 1 convolution, ensuring consistent dimensionality across all feature maps, regardless of their original sizes. The core component of the FFPN module is the focus feature module, which is specifically designed to handle multi-scale features. It processes three different resolution feature maps, performing operations such as up-sampling, down-sampling, and convolution with various kernel sizes. These operations are followed by a series of depth-wise convolutions, which are applied to capture detailed spatial patterns at different scales. The outputs from these convolutions are then aggregated to produce a refined feature representation, which is passed through a final pointwise convolution to generate the final refined features. The process for handling the feature maps is as follows:

(8) $${\bar{x}}_{1}={Conv}_{1}(x_{1}),{\bar{x}}_{2}={Conv}_{2}(x_{2}),{\bar{x}}_{3}=ADown(x_{3})$$

First, the input feature maps are processed individually. The first feature map $x_{1}$ undergoes up-sampling and a 1 × 1 convolution, the second feature map *x*_2_ is processed by a 1 × 1 convolution, and the third feature map $x_{3}$ undergoes the ADown operation, which includes average pooling followed by max pooling and convolution. After these initial steps, the results ${\bar{x}}_{1}$, ${\bar{x}}_{1}$, and ${\bar{x}}_{3}$ are concatenated. The concatenated feature map is then processed by depth-wise convolutions with kernel sizes *k* = {5, 7, 9, 11}, allowing the network to capture multi-scale contextual information more effectively.

(9) $$\begin{array}{c} \bar{x}=Concat\left({\bar{x}}_{1},{\bar{x}}_{2},{\bar{x}}_{3}\right)\\ \;feature=\sum_{k}DWConv_{k}\left(\bar{x}\right) \end{array}$$

The resulting feature map is then processed by a pointwise convolution to aggregate the multi-scale information:

(10) $${\;F}_{r}=PWConv\left(F\right)$$

where *F* denotes the input feature, and *F_r_* represents the refined feature obtained through pointwise convolution.

Finally, the refined feature is added to the original feature map, and a 1 × 1 convolution is applied to produce the final output:

(11) $${\;F}_{out}={Conv}_{1\times 1}(x+F_{r})$$

where *F_out_* denotes the final output feature. Beyond the focus feature module, the overall FFPN architecture consists of two consecutive stages for progressive feature refinement. In the first stage, the projected feature maps are fed into a focus feature module, whose output is further fused with the highest- and lowest-resolution features through down-sampling and up-sampling paths, respectively. These fusion steps are followed by a CSP bottleneck with two convolution blocks(C2f) to enhance contextual understanding [44]. The second stage takes the output of the first stage, along with its fused multi-scale counterparts, and processes them through another focus feature module. The refined output is again passed through top-down and bottom-up pathways, followed by additional C2f blocks. This hierarchical fusion results in final feature maps at three different resolutions, which are then forwarded to the detection head. By combining progressive feature interaction, multi-scale spatial refinement, and lightweight attention via depth-wise convolutions, the FFPN structure significantly enhances the semantic richness and spatial consistency of the intermediate representations.

In this module, the use of depth-wise convolutions and the integration of features at different scales allow for the enhancement of both local details and broader contextual information, ensuring that the FFPN module improves the overall feature representation in the network. With the detailed explanation of the FFPN module that refines multi-scale features, it becomes evident that the quality of feature representations is significantly improved. The refined features produced by FFPN are crucial for enhancing the subsequent operations in the DEIM framework, especially in the context of feature matching.

### 2.4. Learning Strategy

A unified training objective is designed for the proposed framework, consisting of three loss components corresponding to image dehazing, self-supervised pretraining, and detection stages, respectively. These include guided image dehazing with DCF-NET, MIMCL, and DEIM.

#### 2.4.1. Loss of DCF-NET

To optimize the network’s performance, DCFNet employs a multi-scale loss function during training. This combines pixel-level L1 loss, SSIM loss, and FFT. The various loss functions are listed as follows:

(12) $$L_{1}=\frac{1}{N}\sum_{i=1}^{N}|I_{pred}^{i}-I_{target}^{i}|$$

where $I_{pred}^{i}$ denotes the predicted foreground interference picture, $I_{target}^{i}$ denotes the target foreground interference picture, and *N* represents the total number of pixels. In DCFNet, the *L*_1_ loss is mainly used to constrain the pixel-level consistency between the enhanced image and the ground-truth clear image. It directly reduces the differences in brightness, color, and detail between the reconstructed image and the ground-truth image, making the output visually closer to the real clear image. Compared with MSE loss, the *L*_1_ loss is less sensitive to outlier pixels, which makes it more effective in preserving edge and texture details and reducing over-smoothing. In image enhancement, dehazing, and low-light restoration tasks, the $L_{1}$ loss can help the model generate more natural and clearer reconstructed results.

SSIM is a metric used to measure the structural similarity between two images. It considers luminance, contrast, and structural information. Unlike traditional pixel-level difference metrics such as PSNR, SSIM is more consistent with human visual perception and can therefore more accurately reflect the quality of dehazed images, especially in terms of detail preservation and structural consistency.

The calculation of SSIM mainly involves three components: luminance, which measures brightness differences based on the mean intensity of images; contrast, which evaluates contrast differences based on variance; and structure, which measures local structural and texture differences based on covariance.

(13) $$L_{SSIM}\left(I_{x},I_{y}\right)=\frac{\left(2\mu_{x}\mu_{y}+C_{1}\right)\left(2\sigma_{xy}+C_{2}\right)}{\left(\mu_{x}^{2}+\mu_{y}^{2}+C_{1}\right)\left(\sigma_{x}^{2}+\sigma_{y}^{2}+C_{2}\right)}$$

where *μ_x_* and *μ_y_* denote the mean pixel values of the RGB image (*I_x_*) and the processed output image (*I_y_*), respectively. $\sigma_{x}^{2}$ and $\sigma_{y}^{2}$ represent their variances, and $\sigma_{xy}$ denotes the covariance between images x and y. The constants *C*_1_ and *C*_2_ are introduced to avoid division by zero and are usually determined according to the dynamic range *L* of the image. Common default settings are *C*_1_ = (0.01*L*)^2^ and *C*_2_ = (0.03*L*)^2^.

Frequency-domain learning has been widely explored in image restoration because the Fourier transform provides an effective way to model global contextual information. In the frequency domain, low-frequency components mainly describe smooth regions and global illumination, while high-frequency components preserve edges, textures, and fine image details. For the frequency-domain loss, *L_FFT_*, we adopt formula 8 from reference [45] to calculate it. Finally, the total loss is computed across multiple scales.

(14) $$L_{DCFNET}=\sum_{s=1}^{3}\left[\alpha_{1}L_{1}^{s}+\alpha_{2}L_{SSIM}^{s}+\alpha_{3}L_{FFT}^{s}\right]$$

where s represents the different feature map scales [1, 0.5, 0.25], and the coefficients $\alpha_{1}$ = 1.0, $\alpha_{2}$ = 0.5, and $\alpha_{3}$ = 0.1 are empirically determined. In summary, DCFNet effectively combines the advantages of both RGB and YCbCr color spaces, allowing it to restore image details and enhance color perception, making it highly effective for real-world dehazing tasks. This method ensures significant improvement in image quality and provides clearer and more accurate input for subsequent high-level computer vision tasks.

#### 2.4.2. Loss of MIMCL

The model’s primary objective during this phase is to align the output distributions of the teacher and student networks. Specifically, the cross-entropy loss between their class token predictions is minimized, as expressed as follows:

(15) $$L_{cls}=H\left(Z_{cls}^{t},Z_{cls}^{s}\right)$$

where *H*(⋅) represents the cross-entropy loss, and $Z_{cls}^{t}$ and $Z_{cls}^{s}$ are the class token outputs from the teacher and student networks, respectively. For MIM, we incorporate self-distillation techniques, where *M* random mask sequences *m* ∈ {0, 1} are applied to the image. The masked patches are then replaced by a learnable token embedding, resulting in a corrupted image. The teacher network processes the original image, while the student network processes the corrupted image and aims to recover the masked patches. The loss for the masked patches is defined as follows:

(16) $$L_{patch}=\sum_{i=1}^{M}m_{i}\cdot H\left(P_{i}^{t},P_{i}^{s}\right)$$

where $m_{i}$ is the binary mask indicating whether the *i*-th patch is masked, and $P_{i}^{t}$ and $P_{i}^{s}$ are the predictions from the teacher and student networks, respectively. This loss ensures that the student network reconstructs the masked regions by aligning with the teacher network’s output distribution. Additionally, to further enhance image reconstruction, the decoder is tasked with predicting the RGB values of the image. To accomplish this, we use a mean squared error (MSE) loss that compares the predicted RGB features $\hat{Y}$ with the true features Y, as follows:

(17) $$L_{MSE}=\frac{1}{N}\sum_{i=1}^{N}{\left\|Y_{i}-{\hat{Y}}_{i}\right\|}_{2}^{2}$$

where Y and $\hat{Y}$ are the input RGB image features and the predicted RGB image features. This ensures that the decoder not only reconstructs the masked patches but also recovers the overall image’s RGB information. Once the pretraining phase is completed, the model continues to leverage the feature representations learned during this stage, which helps improve its generalization ability. By learning the discriminative features of the components from the contact network, the MIMCL framework significantly enhances the model’s transferability, enabling it to effectively apply the knowledge gained during the pretraining phase to new tasks.

(18) $$L_{mimcl}=L_{cls}+\alpha_{3}L_{patch}+\alpha_{4}L_{MSE}$$

where α_3_ = 0.5 and α_4_ = 2. By jointly optimizing these objectives, MIMCL enables the student network to learn both global semantic consistency and local structural representations from the teacher network. The class-token distillation loss promotes high-level semantic alignment, the patch reconstruction loss strengthens the recovery of masked regions, and the RGB reconstruction loss further improves pixel-level fidelity. As a result, the pretrained model can obtain more robust and transferable feature representations, which provides a stronger initialization for downstream CSC detection tasks under complex railway environments.

#### 2.4.3. Loss of DEIM

The one-to-one (O2O) matching approach, commonly adopted in DETR-based models, pairs each target with exactly one predicted query. This method, which uses the Hungarian algorithm, enables end-to-end training and eliminates the need for NMS. However, a major limitation of this approach is its tendency to produce fewer positive matches compared to traditional one-to-many (O2M) techniques like SimOTA. As a result, supervision becomes sparse, which can slow down training convergence. To address this issue, we introduce Dense O2O, an enhanced strategy that retains the one-to-one matching structure of O2O (with $M_{i}$ = 1) while increasing the number of targets *N* per image to provide denser supervision [46]. Specifically, we divide the original image into four quadrants and merge them into a single image, maintaining the original size. This process boosts the target count from 1 to 4, thereby improving the supervision level as shown in Equation (19), without altering the matching structure. Dense O2O achieves a supervision level comparable to O2M but avoids additional complexity and computational cost.

(19) $$L_{DEIM}=\sum_{i=0}^{N}\sum_{j=0}^{M}F\left(y_{ij},y_{i}\right)$$

where *N* is the total number of targets, *M_i_* is the number of matches for the *i*-th target, $y_{ij}$ represents the *j*-th match for the *i*-th target, *y_i_* denotes the *i*-th ground-truth label, and f is the loss function. Alongside improving matching efficiency, enhancing matching quality is another critical objective in the DEIM framework. Although varifocal loss (VFL) has demonstrated effectiveness in improving detection performance by emphasizing high-confidence predictions, it tends to assign lower weights to low-quality matches, resulting in insufficient supervision for these samples. This imbalance limits the model’s ability to effectively learn from hard or ambiguous predictions. To address this issue, we propose the match ability-aware loss (MAL), which explicitly incorporates match quality into the loss formulation. Unlike VFL, MAL is designed to be more sensitive to low-quality matches by re-weighting supervision according to match ability scores, ensuring that both high- and low-IoU predictions contribute to model optimization. In this way, MAL improves learning stability across different match qualities and enhances overall robustness, particularly for challenging or uncertain predictions. The MAL can be defined as follows:

(20) $$MAL(p,q,y)=\left\{\begin{array}{ll} -q^{\gamma }log(p)-\left(1-q^{\gamma }\right)log(1-p) & ,y=1\\ -p^{\gamma }log(1-p) & ,y=0 \end{array}\right.$$

where *γ* is a tunable parameter that adjusts the loss sensitivity to match quality; the loss focuses more on refining the prediction for high-quality matches (*q*) and penalizes background samples (*q* = 0) by treating them as negative samples, ensuring the model improves both foreground and background match quality.

## 3. Experiments and Result Analysis

### 3.1. Experimental Settings

The operating environment of the experimental is PyTorch 2.3.1, and the operating environment configuration is as follows: (1) Processor: Intel(R) Xeon(R) CPU E5-2697A v4 @ 2.60GHz; (2) Operating Memory: 16G RAM; (3) GPU: NVIDIA RTX 2080TI GPU; (4) Code Operating Environment: Torch = 2.3.1, Python = 3.10, and Ultralytics8.2; and (5) CUDA Version: CUDA 11.6, cuDNN 8.9.7. DCFNet was optimized with an initial learning rate of 3 × 10^−4^ and a weight decay of 5 × 10^−4^. For MIMCL, the learning rate and weight decay were set to 1.5 × 10^−4^ and 1 × 10^−3^, respectively, to ensure stable teacher–student representation alignment during self-supervised pretraining. For DEIM, a layer-wise learning-rate strategy was employed. The base learning rate was set to 1 × 10^−4^, while the learning rates of the pretrained backbone and the encoder–decoder were set to 1 × 10^−5^ and 1 × 10^−4^, respectively. The weight decay of DEIM was set to 5 × 10^−4^. The AdamW optimizer was adopted to train the proposed framework. The batch size was set to 16. DCFNet, MIMCL, and DEIM were trained for 72, 200, and 500 epochs, respectively. For visualization experiments, a confidence threshold of 0.7 was applied only to YOLO-based methods during postprocessing.

### 3.2. Introduction of Dataset

**Figure 5:**
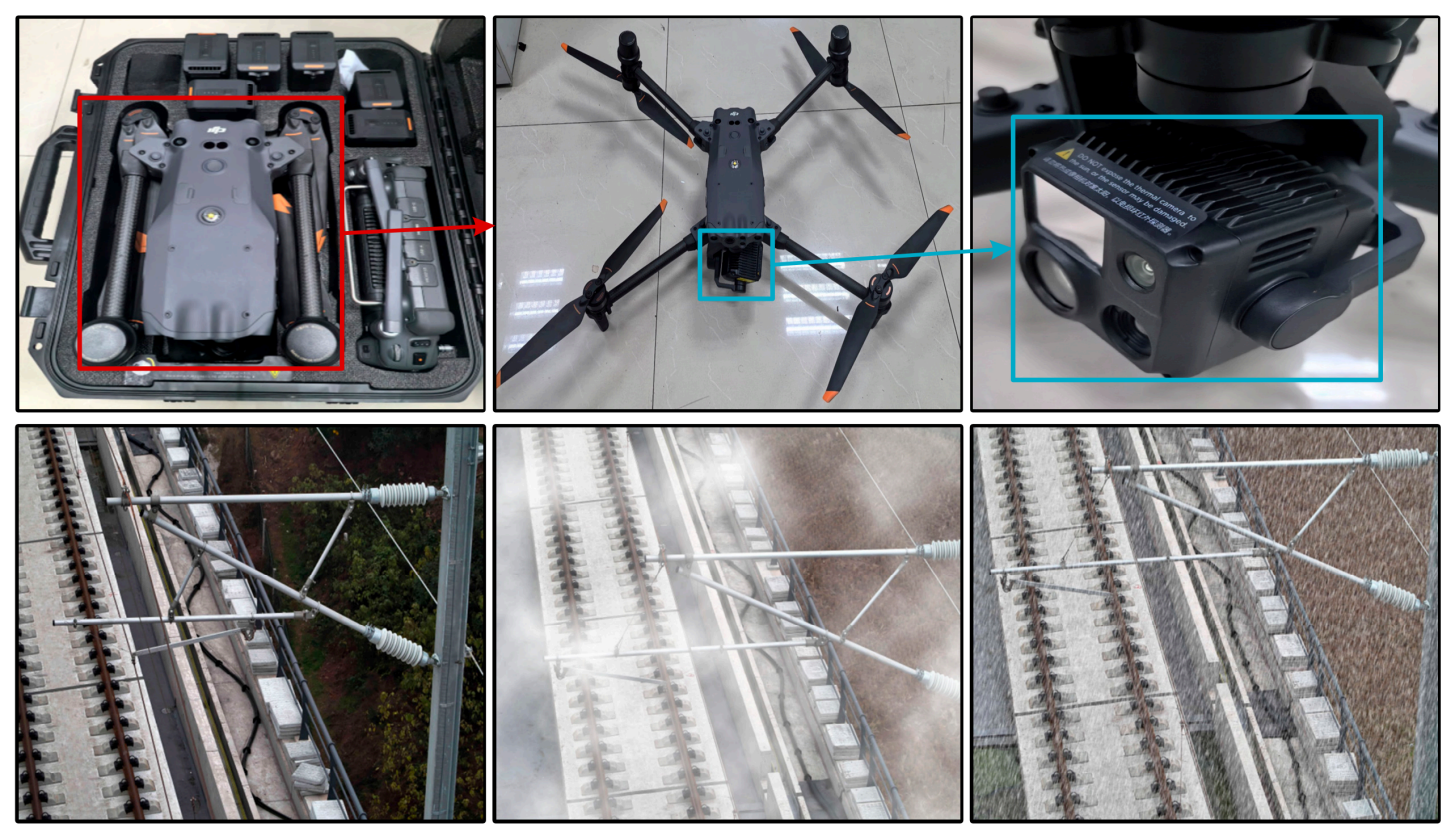
Visualization of the inspection UAV and the collected original, fog, and rain images.

To validate the proposed method, a CSC dataset was created using a UAV. The dataset was gathered with a DJI Matrice M30T UAV (SZ DJI Technology Co., Ltd. Shenzhen, Guangdong, China) while flying over a railway segment. This UAV is equipped with a 480,000-pixel zoom camera, a 120,000-pixel wide-angle camera, a laser rangefinder, and an RTK navigation system that also includes obstacle avoidance. During image collection, the 480,000-pixel zoom camera was used with a 20× zoom for capturing the images. The images were captured at a resolution of 6000 × 8000, which corresponds to 8K resolution. These high-resolution images were taken from approximately 50 m from the contact network using a 20× zoom lens. An image of the UAV is shown in Figure 5.

**Figure 6:**
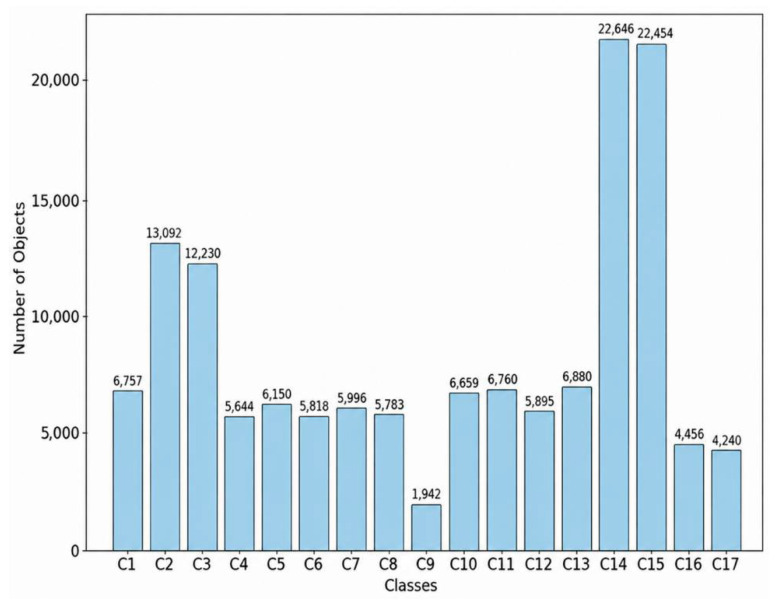
The distribution statistics of UAV datasets.

The dataset includes a total of 5832 UAV cruise images, and this experiment has studied part of the important, electrified railway catenary support components. Specifically, it contains a total of 17 catenary components as shown below: casing base (C1), insulator (C2), insulator base (C3), isoelectric line (C4), load-bearing cable base (C5), locator-bracing base (C6), locator-bracing base ear (C7), locator clamp (C8), locator hook (C9), locator ring (C10), locator tube connector (C11), rectangular locator (C12), rotary double ear (C13), sleeve double ear (C14), sleeve screw (C15), windproof wire (C16), and windproof wire ring (C17). The target detection models are usually trained on clear images. However, in practical applications, contact network images are often affected by adverse conditions such as low light, smog, and heavy rain, which leads to a decrease in detection accuracy. To enhance the robustness of the model in complex working conditions, this study employed algorithms to simulate three typical visual interference environments and generated corresponding enhanced data: (1) Low-Light Environment Simulation: To address the issue of unclear details caused by components in low-light conditions or low luminance, the brightness of the image is adjusted to simulate the change in illumination, enabling the model to adapt to significant variations in light and ensuring that the model can identify and judge components even in dimly lit environments. (2) Haze Environment Simulation: Haze causes images to become blurry and the contrast to decrease, and thus the image with haze (with haze coefficient α) is simulated to different degrees to simulate the interference caused by haze, training the model to adapt to the characteristics of low-contrast features. (3) Heavy Rain Environment Simulation: For rain obscuration and visual noise, visual noise effects such as fog and water droplets are added to the image to simulate the visual effect under such conditions, ensuring the stability of the training model in the visual conditions of a rainy day or fog. Among them, a total of 143,202 component instances are included, and the distribution of each component is shown in Figure 6. We have divided the training and test sets in a 4:1 ratio. Images collected from the same UAV inspection route and adjacent frames are assigned to the same subset to avoid overlap between the training and testing sets. This ensures that images from the same railway segment, viewpoint, or acquisition sequence do not appear in both splits.

### 3.3. Evaluation Indicator

To comprehensively evaluate both the dehazing quality and detection performance of the proposed model, we adopt a set of quantitative metrics. For image quality assessment, PSNR and SSIM are employed as reference-based indicators.

(21) $$\begin{array}{c} PSNR=10\cdot \log_{10}\left(\frac{MAX^{2}}{MSE}\right),\\ MSE=\frac{1}{HW}\sum_{i=1}^{H}\sum_{j=1}^{W}{\left(I_{ij}-K_{ij}\right)}^{2},\\ SSIM(x,y)=\frac{\left(2\mu_{x}\mu_{y}+C_{1}\right)\left(2\sigma_{xy}+C_{2}\right)}{\left(\mu_{x}^{2}+\mu_{y}^{2}+C_{1}\right)\left(\sigma_{x}^{2}+\sigma_{y}^{2}+C_{2}\right)} \end{array}$$

where *MAX* is the maximum possible pixel value (typically 255), *I* is the ground truth image, *K* is the predicted image, and *H* and *W* denote the height and width of the image, respectively. For SSIM, $\mu_{x}$ and $\mu_{y}$ are the means of images *x* and *y*; $\sigma_{2}$, *x*, *σ*, and *y* are their variances; $\sigma_{xy}$ is the covariance; and *C*1 and *C*2 are constants for stability.

### 3.4. Detection Performance Metrics

To reasonably evaluate the detection performance of the model, the following indexes are used in this paper: average precision (AP), mean average precision (mAP), frames per second (FPS), floating-point operations (FLOPs), and parameters (Pa).

(22) $$\begin{array}{c} P=\frac{TP}{TP+FP},R=\frac{TP}{TP+FN}\\ AP=\int \begin{array}{c} 1\\ 0 \end{array}P(R)dR,mAP=\frac{\sum_{q=1}^{Q}AP(q)}{Q} \end{array}$$

where *TP*, *FP*, and *FN* represent the number of true positive samples, false positive samples, and false negative samples, respectively. *P* and *R* represent precision and recall. *AP* is average precision, which is the area of the *PR* curve. *mAP* represents mean average precision, which is the mean value for all catenary components.

### 3.5. Experimental Analysis

(1) **The Analysis of Image Dehazing Methods:** To evaluate the effectiveness of the proposed DCFNET in image dehazing tasks, comparative experiments were conducted using images resized to 640 × 512 for both training and testing. Two image quality metrics—PSNR and SSIM—were adopted for comprehensive evaluation, where higher values of PSNR and SSIM indicate better image quality. As presented in Table 1, DCFNET achieves the best performance across all quality metrics (PSNR: 22.56, SSIM: 0.826), significantly outperforming other methods. Furthermore, DCFNET maintains a lightweight computational footprint (13.32M parameters and 53.40G FLOPs), demonstrating an optimal balance between visual quality and efficiency.

**Figure 7:**
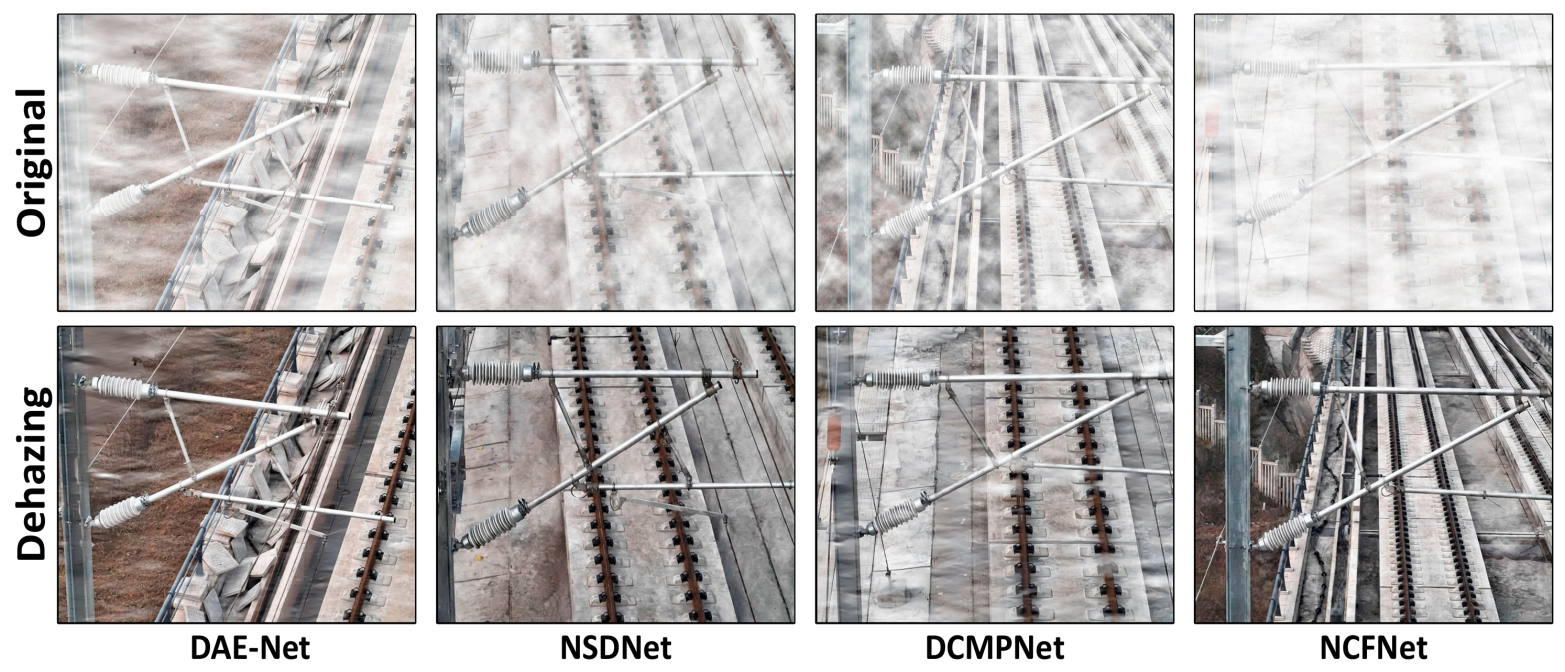
The visual analysis of dehazing results across different methods.

Figure 7 presents a visual comparison of different methods on our UAV-based dataset for image dehazing. Our proposed method demonstrates superior performance compared to other methods. Specifically, DAE-Net improves overall visibility but still leaves residual haze in certain regions. DCMPNet achieves a balanced trade-off between haze removal and texture preservation; however, slight haze remains visible in complex regions. Although NSDNet effectively removes most of the haze, it tends to blur the image and compromise fine details and image contrast. In comparison, DCFNet not only achieves more thorough haze removal but also better preserves natural textures and visual contrast.

(2) **The Analysis of Ablation Study Analysis:** To further validate the effectiveness of the proposed components, we perform ablation studies on MIMCL, FFPN, and Dense O2O. Notably, all ablation experiments are conducted on dehazed images to ensure consistency with the full detection pipeline. The corresponding results are summarized in Table 2. The FPS is measured on input images with a resolution of 640 × 640. An analysis of the results shown in the ablation table indicates the following findings: (1) The integration of MIMCL, FFPN, and Dense O2O each contributes to noticeable improvements in detection accuracy. (2) Specifically, MIMCL alone improves *mAP*_50–95_ from 0.652 to 0.703, while FFPN and Dense O2O individually raise it to 0.671 and 0.683, respectively. When combining MIMCL with FFPN or Dense O2O, the *mAP*_50–95_ further increases to 0.734 and 0.727. Upon enabling all modules, the best performance is achieved with an *mAP*_50–95_ of 0.755, an *mAP50* of 0.992, and a *mAR* of 0.991. (3) In terms of inference speed, the model maintains real-time performance, achieving 20.9 FPS even when all modules are enabled. The additional computational cost introduced by each component is minimal. These results demonstrate that the proposed modules are complementary and effective, striking a favorable balance between detection accuracy and speed.

**Figure 8:**
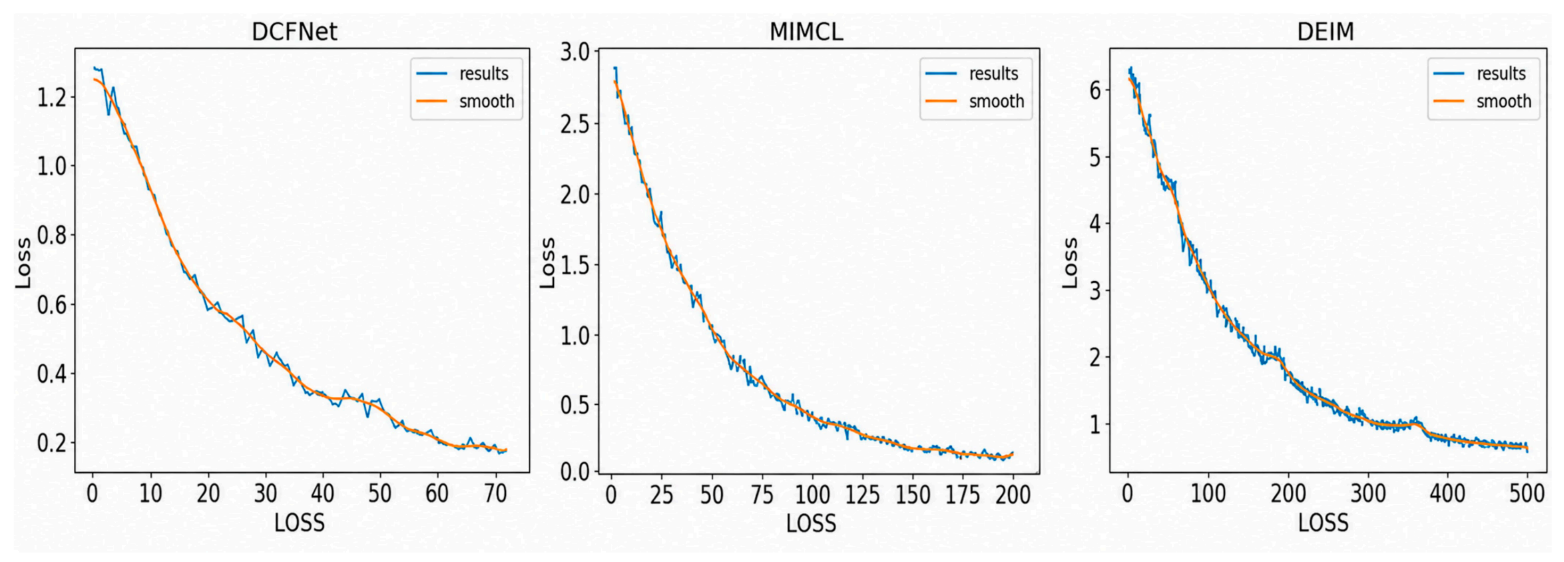
Training convergence of the proposed modules based on total loss.

Figure 8 shows the total loss curves of DCFNet, MIMCL, and DEIM during training. The blue curves represent the original training results, while the orange curves denote the smoothed loss trends. As training progresses, the total losses of all three modules decrease steadily and gradually converge, indicating stable optimization. DCFNet converges rapidly within a relatively short training schedule, demonstrating effective learning of low-level restoration features. MIMCL shows a smooth and continuous decline, suggesting stable self-supervised representation learning. DEIM is trained for a longer period, and its loss gradually stabilizes, indicating effective optimization of the detection objective. Overall, the convergence behavior confirms that the proposed loss functions can effectively guide the training of different modules.

(3) **The Analysis of Self-Supervised Learning:** It is also important to note that all subsequent experiments are conducted on dehazed images to ensure consistency with real-world UAV inspection scenarios. The evaluation results of the network utilizing different pre-trained weights from various self-supervised learning methods are presented in Table 3. Clearly, our proposed method, MIMCL, consistently outperforms comparative self-supervised methods across multiple critical metrics. Specifically, MIMCL achieves the highest *mAP* of 75.53%, *AP*_50_ of 99.27%, and ${AP}_{75}$ of 80.23%. In comparison, other self-supervised methods such as MAE, IGPT, SimMIM, BEIT, and DINO achieve relatively lower performance. For example, MAE achieves a *mAP* of 67.91%, SimMIM reaches 72.18%, and BEIT attains 74.31%. Particularly notable is MIMCL’s significant improvement in *mAP* and AP_75_, surpassing baseline methods by approximately 1.22–7.62%. These results demonstrate that our proposed MIMCL framework effectively leverages self-supervised strategies, leading to enhanced feature representation capabilities and improved detection performance.

**Figure 9:**
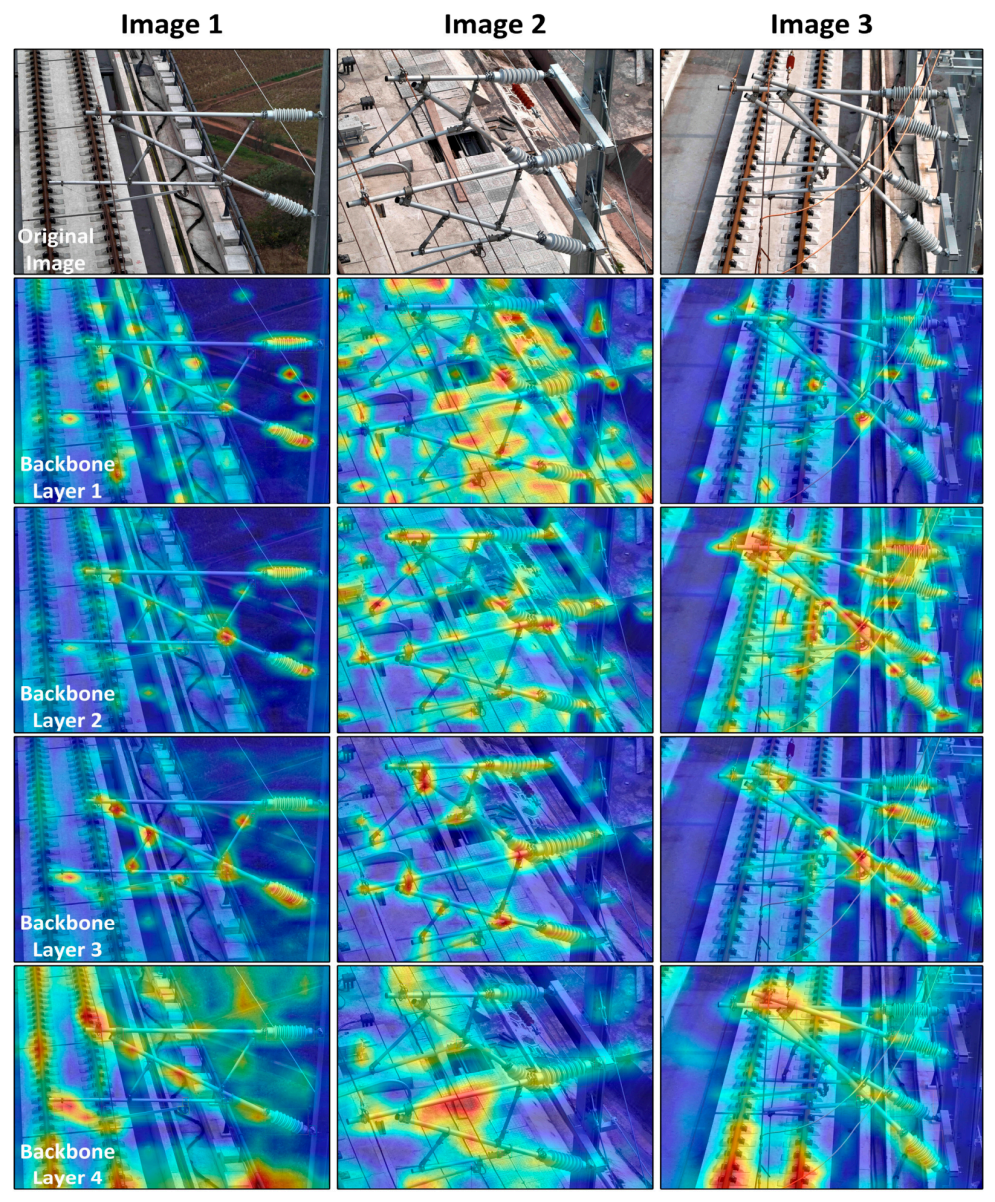
The visualization of the heatmap in different layers.

To validate the application of prior knowledge learned through MIMCL, heatmap visualization analysis, as shown in Figure 9, reveals that with the deeper layers of the network, the model increasingly focuses on the regions corresponding to the catenary components. The heatmap colors range from blue (low attention) to yellow and red (high attention), indicating that deeper layers pay more attention to these critical areas. This demonstrates that the prior feature information learned during self-supervised training is successfully integrated into the backbone network, leading to improved detection performance. This further and more intuitively validates the effectiveness and rationality of the MIMCL method.

**Figure 10:**
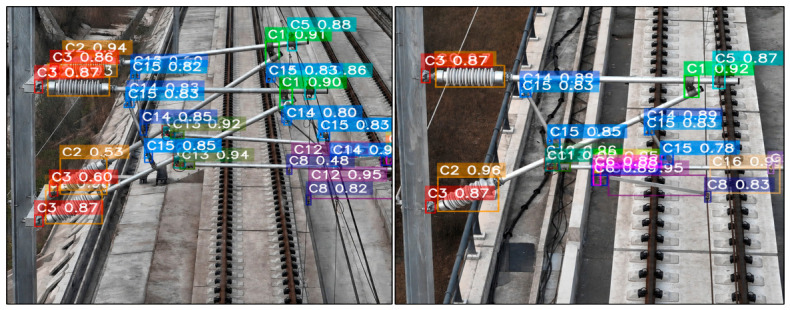
The visual analysis of FF-DEIM.

**Figure 11:**
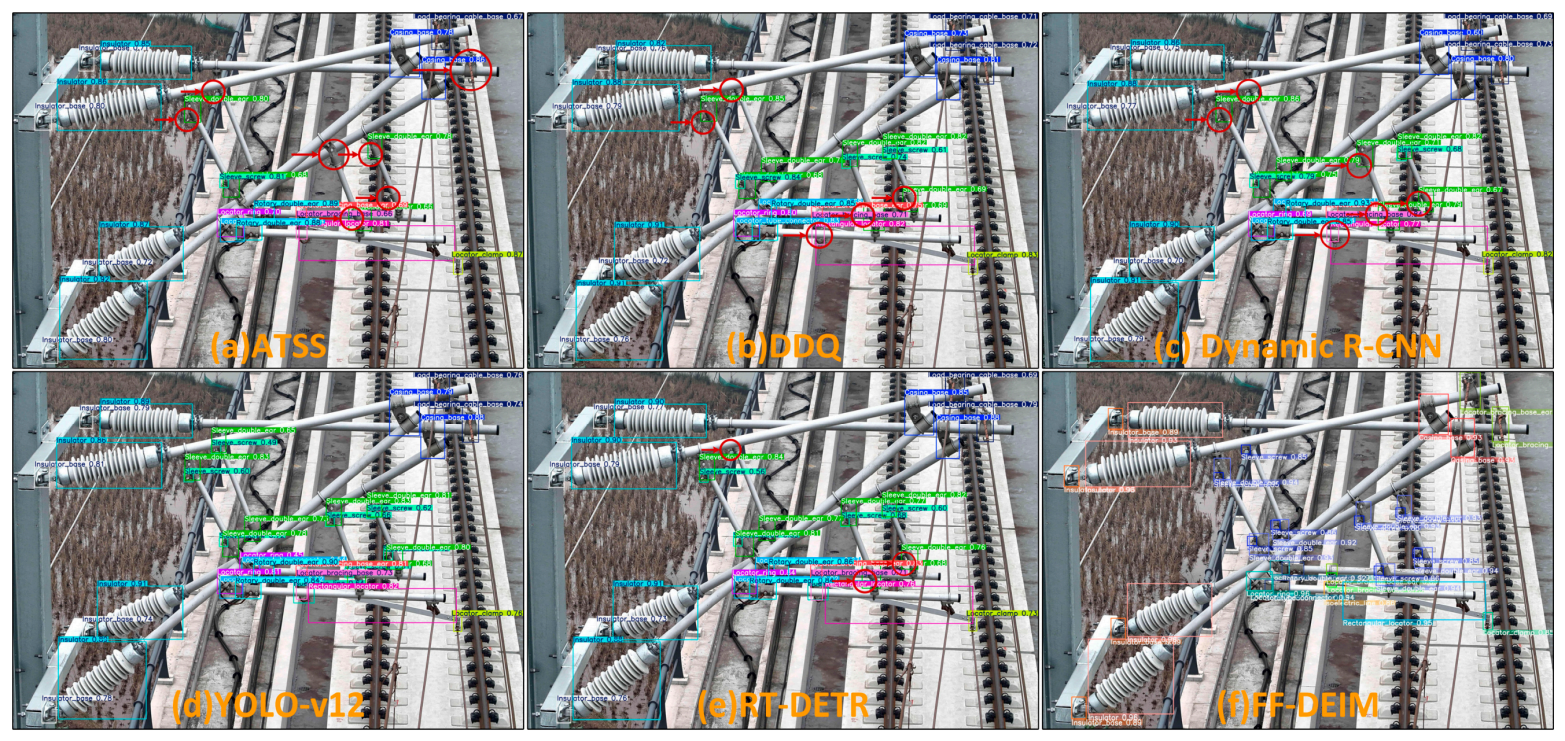
The visual analysis with different methods.

(4) **The Analysis of Multi-Scale Feature Fusion Module:** By analyzing the results in Table 4, we observe the following findings: (1) Our proposed metised hod, FFPN, achieves the best overall detection performance, with an *mAP* of 67.43%, an *AP*_50_ of 95.54%, an *AP*_75_ of 75.26%, and an *AP_s_* of 65.88%, demonstrating its strong ability to handle both small and large object detection tasks. (2) The CFP method performs well, reaching the highest performance on *AP75* and *APl*, with values of 73.96% and 83.95%, respectively, benefiting from its cross-feature pyramid design that helps preserve local details in high-level features. (3) CR-FPN and Bi-FPN show comparable results, but FFPN slightly outperforms them, especially in *mAP* and *AP75*, highlighting the effectiveness of FFPN in aggregating multi-level features and improving robustness in complex environments. At the core of FFPN is the focus feature module, which introduces a more focused and detailed approach to multi-scale feature handling. By refining features at multiple scales and capturing spatial patterns at different granularities, the focus feature module enhances the model’s ability to handle complex, multi-scale objects and improve overall detection performance. This focus on detailed feature refinement makes FFPN particularly effective in scenarios where object size and scale vary significantly, outperforming other FPN variations.

(5) **The Analysis of Classic Detection Methods**: Following the FPN experiments, we further evaluated the performance of our method on object detection tasks. Specifically, we conducted comparative experiments with several classical object detection approaches, as shown in Table 5. Both training and testing images were resized to 640 × 640. The comparison experiments were structured as follows: (1) For one-stage detectors, YOLOv11-l, YOLOv12-l, and ATSS served as baseline methods. (2) For two-stage detectors, Cascade R-CNN and Dynamic R-CNN were selected as comparison methods. (3) Among state-of-the-art DETR-based approaches, Align-DETR, DDQ (DETR), DINO, HDINO, and RT-DETR were also included in the evaluations. As demonstrated in Table 5, the proposed method achieves an excellent balance between detection accuracy and inference speed, highlighting its advantages as follows: (1) Our proposed method achieves optimal performance across multiple detection metrics, obtaining *mAP*_50–95_ = 75.53%, *AP*_50_ = 99.27%, *AP*_75_ = 80.23%, *APs* = 65.71%, and *APl* = 85.34%, while maintaining a competitive inference speed (20.9 FPS). (2) Notably, in small-object detection, our method achieves APs = 65.71%, clearly outperforming representative models such as YOLOv11-L (63.53%), YOLOv12-L (63.98%), and RT-DETR (62.01%), demonstrating superior effectiveness in challenging small-scale scenarios. (3) Although YOLO-series methods (YOLOv11-L and YOLOv12-L) achieve higher inference speeds (32.6 FPS and 30.7 FPS, respectively), they show significantly lower overall detection accuracy and small-object performance compared to our method. (4) When compared with RT-DETR, our method not only increases *mAP*_50–95_ by approximately 10.26% but also significantly enhances small-object detection accuracy at similar inference speeds, further underscoring the efficiency of our designed modules. In summary, the proposed method effectively balances detection accuracy, especially for small-scale objects, and inference speed, providing clear advantages over both YOLO-series and DETR models.

(6) **Visualization of Detection Results**: To further verify the practical effectiveness of the proposed method, object detection experiments were conducted on the dehazed images. As shown in Figure 10, our method is capable of accurately detecting components with significant scale variations, such as insulators (within insulator groups) and screws (support sleeve screws). This clearly demonstrates the adaptability and robustness of our method in addressing complex backgrounds and multi-scale challenges. To further validate the superiority of the proposed method, we conducted additional visualization experiments using six different detection methods. As shown in Figure 10, the letters (a) to (f) correspond to the detection results of the ATSS, DDQ, Dynamic R-CNN, YOLOv12, RT-DETR, and FF-DEIM detectors on the same image. In this experiment, we selected the detector with the best overall performance from various methods. Red arrows in the figure indicate the areas where catenary components were missing by the detectors. Based on the results, we can make the following observations: (1) the ATSS detector (a) failed to detect multiple components, including four sleeve screws, a load-bearing cable base, a sleeve double ear, and an isoelectric line; (2) the DDQ (b) and Dynamic R-CNN (c) detectors exhibited similar performance, each missing four sleeve screws and one isoelectric line; (3) RT-DETR (e) also showed some deficiency, with three sleeve screws left undetected; and (4) in contrast, both YOLOv12 (d) and FF-DEIM (f) demonstrated complete detection coverage, successfully identifying all catenary support components without any omissions. As shown in Figure 11, the proposed method achieves accurate detection of components with significant scale variations, such as insulators within insulator groups and support sleeve screws. The results demonstrate the robustness of the method under complex backgrounds and multi-scale scenarios.

## 4. Conclusions

In summary, we propose an efficient method for detecting catenary support components, integrating multiple innovative strategies. The contributions of our method are as follows: (1) DCFNet enhances image quality by removing haze, improving detection accuracy; (2) MIMCL pretraining improves feature representation, enabling the model to focus on small-scale components and enhancing performance in complex scenarios; (3) in the FF-DEIM framework, FFPN refines multi-scale feature fusion, improving feature quality by integrating features at different scales, which enhances detection accuracy, especially for small-scale and complex objects. Additionally, Dense O2O improves supervision by increasing the number of targets per image, providing denser supervision and accelerating training convergence without adding complexity. Finally, we draw the following conclusions from the comparative experiments and visual analysis: (1) The proposed DCFNet method significantly enhances image quality by removing haze while preserving fine details, leading to better detection performance. (2) The integration of MIMCL pretraining improves feature representation, enabling the model to focus on key components, particularly small-scale catenary components, which enhances overall detection accuracy. (3) The FFPN module improves multi-scale feature fusion, refining feature representations at different scales, which enhances the model’s ability to handle both small and large objects effectively. (4) The combination of FFPN with Dense O2O improves the localization accuracy, providing denser supervision and accelerating training convergence without additional complexity, leading to better detection of catenary components. In future research, we will combine image dehazing techniques with anomaly detection methods to enhance the detection of irregularities in catenary components, particularly in challenging environmental conditions.

## Figures and Tables

**Table 1:** Comparative experiments for image dehazing methods.

Methods	PSNR	SSIM	Params/M	FLOPS/G
MB-Talyor [47]	19.52	0.605	7.43	44.05
NSDNet [48]	21.83	0.611	11.38	56.86
DAE-Net [49]	21.22	0.574	3.65	32.23
OKNet [50]	21.43	0.634	4.72	39.67
DCMPNet [51]	19.93	0.597	7.16	62.89
DCFNet (Ours)	22.56	0.826	13.32	53.40

**Table 2:** The results of ablation experiments.

Module			Accuracy			Speed
MIMCL	FFPN	Dense O2O	*mAP*_50_	*mAP*_50–95_	*mAR*	*FPS*
			0.979	0.652	0.971	24.1
**✓**			0.987	0.703	0.983	23.5
	**✓**		0.982	0.674	0.974	23.8
		**✓**	0.984	0.683	0.976	22.6
**✓**		**✓**	0.989	0.727	0.987	21.8
**✓**	**✓**		0.990	0.734	0.988	22.2
**✓**	**✓**	**✓**	0.992	0.755	0.991	20.9

**Table 3:** Comparative experiments for self-supervised learning methods.

SL	*mAP*	*AP*_50_	*AP*_75_	*AP_s_*	*AP_m_*	*AP_l_*
MAE [52]	67.91	96.44	77.9	62.61	72.64	82.55
IGPT [53]	71.98	96.94	77.54	65.51	73.08	82.92
SimMIM [42]	72.18	96.57	77.34	64.62	72.42	82.76
BEIT [54]	74.31	97.33	78.51	65.68	**74.29**	83.54
DINO [55]	73.32	98.23	78.31	65.54	73.47	83.11
MIMCL (ours)	**75.53**	**99.27**	**80.23**	**65.71**	72.32	**87.34**

**Table 4:** Analysis of feature pyramid network.

Methods	*mAP*	*AP*_50_	*AP*_75_	*AP_s_*	*AP_m_*	*AP_l_*
FPN	60.41	94.28	67.33	59.24	70.62	81.39
PA-FPN [56]	61.46	93.21	67.76	60.96	69.95	80.07
NAS-BFPN [57]	61.73	94.01	68.16	60.59	68.61	80.72
CR-FPN [58]	62.47	93.93	68.84	60.71	71.03	81.39
CFP [59]	65.45	94.81	73.96	64.32	72.46	**83.95**
FFPN (ours)	**67.43**	**95.54**	**75.26**	**65.88**	**75.08**	83.07

**Table 5:** Comparative experiments for classic detection methods.

Methods	*mAP*	*AP*_50_	*AP*_75_	*AP_s_*	*AP_m_*	*AP_l_*	Pa/M	FLOPs/G	FPS
Dynamic RCNN	58.33	91.83	62.58	56.97	62.63	78.17	41.41	197.7	16.2
Cascade RCNN [60]	56.87	91.41	62.63	53.16	62.37	76.58	69.19	225.4	14.8
ATSS [61]	57.73	92.93	61.85	56.96	65.51	77.69	32.14	192.4	15.6
DINO [62]	62.87	94.08	68.93	61.58	71.58	81.92	47.56	265.6	15.5
Align-DETR [63]	64.25	94.58	71.58	60.83	70.13	81.54	47.51	253.8	15.1
HDINO [64]	63.76	94.10	70.78	60.12	69.49	80.83	68.10	281.4	16.5
DDQ (DETR) [65]	63.44	94.18	71.47	61.25	70.42	82.42	65.75	852.5	15.4
YOLOv11-L [66]	66.28	95.21	75.24	63.53	73.24	83.43	**25.32**	**87.3**	**32.6**
YOLOv12-L [67]	67.74	94.79	73.57	63.98	**73.82**	83.38	26.42	89.5	30.7
RT-DETR [68]	65.27	97.92	72.29	62.01	73.06	82.84	42.14	125.7	23.2
FF-DEIM (ours)	**75.53**	**99.27**	**80.23**	**65.71**	72.32	**85.34**	67.35	153.3	20.9

## Data Availability

The data presented in this study are not publicly available due to confidentiality restrictions. Access to the data is restricted to the research team and authorized personnel only. Further inquiries can be directed to the corresponding author.
